# Impact of lab-based drug checking services on syringe service program engagement in Maryland, USA

**DOI:** 10.1186/s12954-025-01380-z

**Published:** 2026-03-05

**Authors:** Caitlin M. Berry, David Newton, Margaret Rybak, Gregory Malik Burnett, Marcela Najarro, Edward Sisco, Jasmine Lopes, Dennis Leber

**Affiliations:** 1 Statistical Engineering Division, National Institute of Standards and Technology, 100 Bureau Drive, Gaithersburg, MD 20899 USA; 2 Materials Measurement Science Division, National Institute of Standards and Technology, 100 Bureau Drive, Gaithersburg, MD 20899 USA; 3 Office of Harm Reduction, Maryland Department of Health, Behavioral Health Administration, 1223 West Pratt Street, Baltimore, MD 21223 USA; 4 Center for Addiction Medicine, University of Maryland Medical Center Midtown Campus, 827 Linden Avenue, Baltimore, MD 21201 USA

**Keywords:** Syringe service program, Overdose, Prevention, Harm reduction, Drug checking

## Abstract

**Purpose:**

The introduction of synthetic substances into the unregulated drug supply has driven a surge in overdose deaths, posing significant public health challenges in timely substance identification, engagement with people who use drugs (PWUD), and access to treatment. Maryland’s rapid analysis of drugs (RAD) program, piloted through syringe service programs (SSPs), provides a proactive harm reduction strategy by integrating mail-in, laboratory based drug checking with broader public health services to enhance surveillance and improve engagement with PWUD. We aim to investigate the association of offering RAD drug checking with SSP metrics such as encounters with PWUD and access to wound care.

**Methods:**

To investigate the association of RAD affiliation with SSP engagement and service metrics, a hierarchical linear model was employed to estimate percent changes in metrics such as encounters, naloxone distribution, and instances of overdose education. Metrics were analyzed on a log scale to account for skewness and variability across sites, with site-specific effects and random variation modeled to capture both baseline differences and the impact of RAD affiliation.

**Results:**

Sites that became RAD affiliated saw an increase in many of the recorded metrics, such as wound care services per quarter (131%) and the number of participants per quarter (74%) while other metrics, such as testing, showed smaller changes. When adjusting for baseline trends of non-RAD affiliated behavior, most of the metrics still trend towards a positive association with RAD affiliation, but only Wound Care and Counseling reach statistical significance given the small number of sites remaining non-RAD affiliated for the full duration of data collection.

**Conclusion:**

Participation in the RAD program shows a positive association with an increase in engagement and service delivery within Maryland’s SSPs, suggesting it may be considered as a valuable public health intervention for improving outcomes for PWUD. However, further research is needed to explore causality and influence of factors like broader public health initiatives.

## Introduction

The emergence of synthetic substances in the unregulated drug supply and the corresponding increase in overdose morbidity and mortality has been well documented in the scientific and public health literature [1–3]. People who use drugs (PWUD) in the state of Maryland, United States (USA) (henceforth referred to as “Maryland” for ease of readability) have experienced this phenomenon first hand with overdose deaths increasing 18% from 2019 to 2020, reaching 2,799 deaths in 2020 [4]. The trend continued into 2021, with illicitly manufactured fentanyl accounting for 84% of all overdose deaths statewide. Numerous challenges exist in the effort to muster a public health response to this overdose epidemic including the timely identification of potent substances in the drug supply [5], identifying and engaging with PWUD to inform them of these dangers [6], and helping these populations navigate the health system to gain access to treatment [7]. Maryland faced similar challenges in its initial response to increasing overdose deaths. Retrospective drug toxicology data from the Office of the Chief Medical Examiner commonly took at least three months to be released to the public, and engagement with PWUD outside the traditional medical system was limited. Interactions with PWUD were often stigmatized, and interventions tended to focus on managing medical complications from chronic drug exposure, complicating efforts to identify the harmful substances responsible for overdoses.

Drug checking has emerged as a promising harm reduction practice in the fentanyl era to help quickly identify novel psychoactive substances introduced into the unregulated drug supply [8–11]. Drug checking is generally implemented with the goal of reducing harm for PWUD by analyzing drug samples directly from the public and returning results to the service user with information on what substances may be present in their samples. This involves an information exchange between the service user and the drug checking service, while carrying out appropriate interventions with the service user. Numerous types of drug checking services have been implemented to help at the point of use for PWUD, including fentanyl and xylazine test strips, which have shown promise in increasing the agency of PWUD to engage in safer use [12]. In Maryland these harm reduction tools have been widely disseminated across the state, however the need to have a robust public health surveillance system for the proactive and rapid identification of novel substances emerging in the unregulated drug supply remains.

In an effort to address this need, the Maryland Department of Health’s Center for Harm Reduction Services (CHRS), the Baltimore-Washington High Intensity Drug Trafficking Area (HIDTA), and the National Institute of Standards and Technology (NIST) initiated a collaborative effort to develop a statewide drug checking program.^1^ The Rapid Analysis of Drugs (RAD) program is the public health component of Maryland’s statewide drug checking initiative. The program is one of the services offered at Syringe Service Programs (SSPs) available across the state. SSPs are a public health strategy centered around drug user health with the primary goal of reducing risk associated with drug use. In Maryland, SSPs were statutorily initiated in 2016, prior to which the Baltimore City Needle Exchange, established in 1994, was the only program offering syringe services. Program staff offer access to clean syringes and other drug paraphernalia, access to medical services for infectious disease and substance use disorder, provide overdose prevention education including access to naloxone, and offer peer recovery support services to help with health system and social safety net navigation. Additionally, state law allows SSP staff and participants to possess drug paraphernalia as part of the program. These programs have been demonstrated to increase trust within communities of PWUD as well as increase engagement with the treatment system, making them a perfect fit to pilot a program designed to directly engage people who use drugs [13].

Beginning in October 2021, RAD was piloted in eight SSPs in various jurisdictions throughout Maryland. Through RAD, staff are able to test paraphernalia provided by SSP participants. Sampling is confidential and no personally identifiable information is shared with CHRS or NIST. Samples are sent to NIST for comprehensive chemical analysis, and results are reported back to the program in near real time, the time-to-result once the lab receives the sample being about 48 h [14]. SSP staff are then required to provide the data back to the person who submitted the sample, typically within 2 weeks. See [14, 15] for a complete timeline and sampling protocol for RAD. Many SSPs also include aggregate data from their program’s sampling in public message boards, handouts, and conversations in order to spread awareness of real-time drug market changes. In October of 2022, RAD expanded to be available for all SSPs. While SSPs are not required to participate in RAD, since October 2022, 16 of Maryland’s 24 SSPs have begun offering RAD drug checking services. The most common barriers for RAD uptake are staff capacity and political resistance.

As mentioned, RAD is only one of many services that SSPs may provide for participants. Since participation in RAD is optional, a natural question of interest is: do we see a meaningful change in the use of other services for those SSPs who participate in RAD? To test the hypothesis that taking part in the RAD program is linked with increased engagement in other services, we examine the possible association RAD participation has with particular metrics collected by a set of SSPs in the state of Maryland, USA. This analysis is an effort to understand how RAD has potentially impacted SSP participation and PWUD in Maryland, which has not been studied in other scientific literature.

## Data

The data for this study focuses on the change in engagement at SSPs who participate in RAD. Since the expansion of RAD, post-pilot study, the majority of SSPs in Maryland have chosen to take part. This is most commonly due to anecdotal benefits of participating, which they hear from other programs. All SSPs are required to submit a standard quarterly report with all metrics reported based on SSP statute (codified in MD Health-General §24-901-909 and regulated under COMAR 10.52.01.01–10.52.01.09) submitted in Cognito Forms [16, 17]. Participation in RAD has no effect on documentation requirements or quarterly reporting expectations. The collected data consists of 24 unique SSP sites located in 13 distinct Maryland counties with measurements taken quarterly from calendar year Quarter 1 (Q1) 2021 to Q1 2024. The dataset also includes descriptive data about each site including county location, urban/suburban/rural designation, date founded as an SSP, and date of transition to RAD, if applicable. For each site, we also have count data (by quarter) of each of the metrics described in Table 1.

**Table 1:** Metric names and descriptions

Metric	Description
Counseling	The number of encounters where a direct service or referral to substance-related disorder counseling, treatment, or recovery was provided
Doses of naloxone distributed	The number of doses of naloxone distributed
Number of encounters	The number of total encounters an SSP has made during the reporting period. This number may count the same individual multiple times, and may include both registered and unregistered individuals. This number may include outreach encounters with individuals who did not access services on site
Number of new participants registered	The number of individuals who registered/enrolled with an SSP during this reporting period. These are participants who received a unique ID and participant ID card for the first time during the reporting period
OD education	The number of encounters where overdose or naloxone use education was given
Reproductive	The number of direct services or referrals to reproductive care
Testing	The number of direct services or referrals to sexually transmitted infection (STI) testing. This is commonly HIV and Hepatitis C testing. For direct services, some programs have onsite testing capabilities, some only provide a rapid test
Number of unique participants	The number of registered individual (unduplicated) participants that have been served by an SSP during this fiscal year. Registered participants who receive services multiples times during the fiscal year are counted once in this measure
Wound care	The number of direct or referral services for wound care. For direct services this may be giving the participant wound care supplies or providing hands-on services

**Fig. 1:**
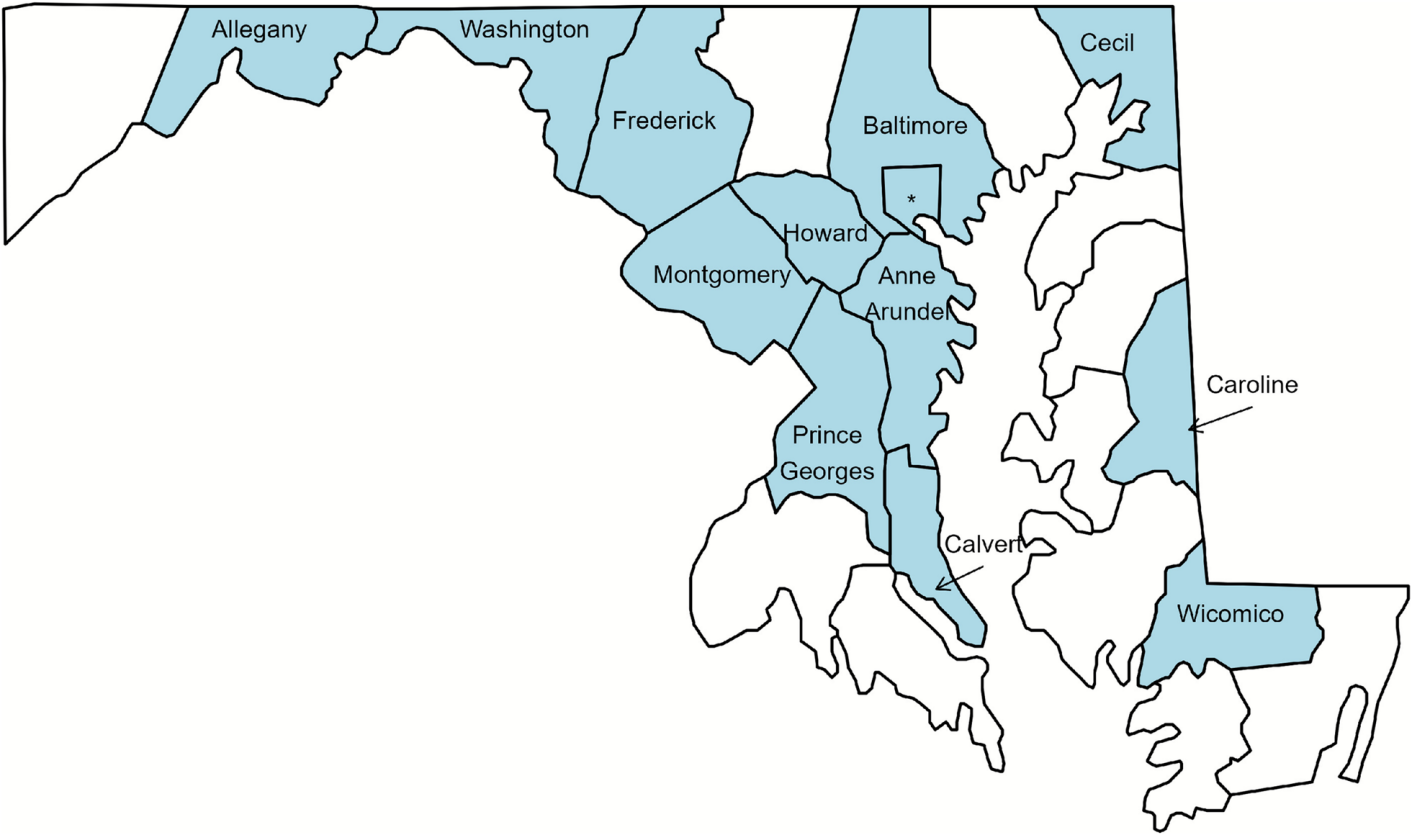
Map of Maryland counties. Counties with SSPs included in study are shown in blue. The * indicates Baltimore City County

The counties served by these sites are shown in the map in Fig. 1. Of the 24 SSPs, 16 began offering RAD testing at some point in 2021-2024 and eight did not offer RAD testing at any point during this time frame. Several data points were excluded from analysis. For sites that only had a single quarter prior to, or after, RAD affiliation, the values from that quarter were omitted. Although the statistical modeling approach does take the duration of RAD affiliation into account, several of the single-quarter values were at extremes, and we did not want these single values to have a disproportionate effect on the results. Specifically, this resulted in dropping data from Q3 of 2022 for the HARP UMD site and data from Q3 of 2021 for the Voices of Hope, Inc. site for several metrics. These represented instances where a site only had a single quarter’s worth of data prior to becoming RAD affiliated. Only the particular quarters’ data were dropped; the rest of the data from these sites for subsequent quarters was kept. Additionally, Q4 of 2023 was dropped for Gaudenzia, Inc. for Wound Care, as this was the only quarter that this site recorded a value for Wound Care (the reported value was “$<11$"). We also excluded the pre-RAD data from the AHEC West site, since they immediately began sampling for RAD upon becoming an authorized SSP, and the increases associated with RAD appeared too large to be realistic. One additional feature of the data was that any value under 11 was indicated as “$<11$," so the exact value is unknown. To minimize the influence of these low values (as we analyzed the data on a log scale), any value recorded as “$<11$” was set to be 10 as a conservative placeholder. To verify that each of these data processing choices did not cause any meaningful change in the analysis, we ran the approach outlined in Sect. 3 with various combinations of these options. The data processing decisions described above generally resulted in more conservative estimates of the percent change in each metric associated with RAD affiliation, and did not significantly affect the analysis.

## Statistical methods

To analyze effects associated with the RAD program, we investigated changes in various metrics associated with a site becoming RAD affiliated. These metrics include information related to traffic occurring at each site, such as the total number of encounters each quarter, as well as health services provided or referred at each site, such as the number of naloxone doses distributed and the number of visitors given resources on overdose (OD) education. A full list of metrics and descriptions can be found in Table 1. For each variable of interest, we estimated the overall percent change associated with RAD affiliation. We modeled the data generation process with a hierarchical linear model, which accounts for site-to-site variation in both the effect of the RAD program as well as the baseline value of each metric.

**Fig. 2:**
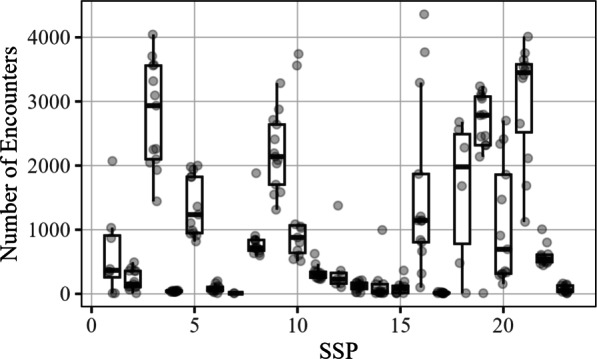
Number of Encounters for each site. Each data point represents the number of total visitors (y-axis) for a specific quarter, at the given site (x-axis)

Traffic at different SSPs spans different orders of magnitude due to differences in geographic region, jurisdiction population, accessibility, and community stigma. As a result, the metrics on the raw scale tend to be strongly skewed. For example, in Fig. 2 below, the majority of SSPs had a median number of quarterly encounters under 500, yet several SSPs saw traffic well into the thousands. Since the different SSPs experience vastly different amounts of traffic, we analyze the data on a log scale. As a consequence of this, our model will estimate the typical relative change associated with a site becoming RAD-affiliated (specifically in terms of the log ratio), as opposed to the absolute change. The statistical model is as follows:

1 $$\begin{aligned} \log (Y_{{ij}} ) = & \,\beta _{0} + \beta _{1} I({\mathrm{rad}}_{{ij}} ) + b_{{0i}} + b_{{1i}} I({\mathrm{rad}}_{{ij}} ) + \epsilon _{{ij}} \\ & \, {\text{for }}i = 1,...,N_{i} ;\;j = 1,...,N_{j} . \\ \end{aligned} $$

Above, $Y_{ij}$ is a random variable representing the $j^{th}$ observation at site *i* for a given metric (e.g., number of encounters), $\beta _0$ represents the underlying mean value of the metric without RAD affiliation, and $\beta _1$ represents the underlying mean change due to RAD affiliation across all sites, which is the primary parameter of interest. The term $I(\text {rad}_{ij})$ is a binary value indicating whether or not the observed response occurred during RAD affiliation or not (yes = 1; no = 0), and $b_0$ and $b_1$ represent site-specific effects for the metrics prior to, and during RAD affiliation, respectively. The $\epsilon _{ij}$ term represents the typical error in measurement due to random variation within a site. The random terms $b_{0i}$, $b_{1i}$, and $\epsilon _{ij}$ are all assumed to be mean-zero Gaussian random variables with their own variance terms to be estimated.

To carry out statistical inference, we fit the model using restricted maximum likelihood using the lme4 R package [18]. Confidence intervals are computed using profile likelihoods.

## Results

**Fig. 3:**
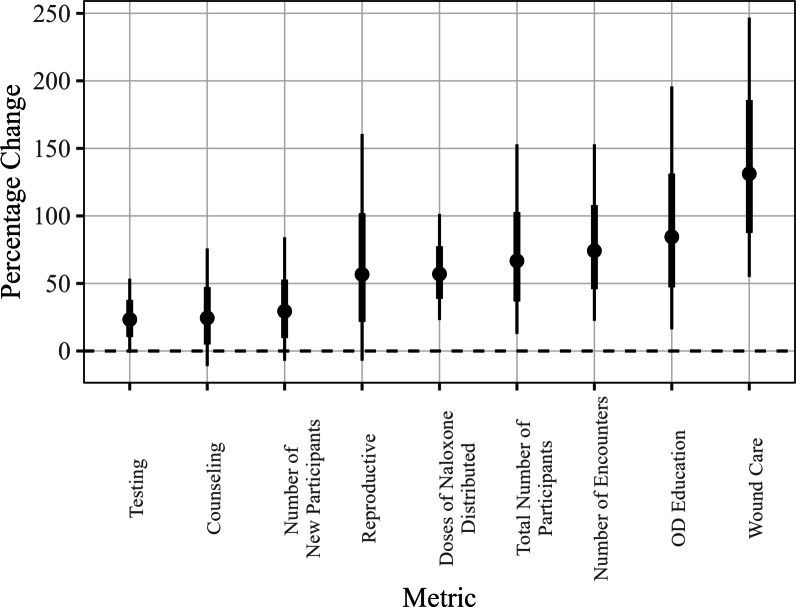
Estimated percent change for all metrics (points), as well as standard errors (thick black lines) and 95% confidence intervals (thin black lines)

Estimates, 95% confidence intervals, and associated p-values for the percent change associated with RAD affiliation are presented in Fig. 3 and Table 2 below for each of the 10 metrics of interest. The Benjamini-Hochberg procedure was used to adjust the p-values to account for multiple testing. The confidence intervals are marginal (unadjusted) intervals derived using profile likelihoods. Testing, counseling, and the number of new participants registered appeared to show small to moderate changes associated with RAD affiliation, with increases estimated in the range of 20% to 30%. For the remaining metrics, there was at least a 50% or greater estimated change, up to a substantial 131% increase for wound care services per quarter. We note that the estimated percent changes have wide ranges for their associated confidence intervals, which is due to natural variation in the data as well as sample size constraints. For example, although the estimated change in the number of encounters is around 74%, the true change could be as low as 22%, or as high as 153%, according to this approach at a level of 95% confidence.

**Table 2:** Estimated effects for all metrics, in terms of percent change, as well as 95% confidence intervals. Starred values indicate those which are statistically significantly different from 0 at a level of $\alpha =0.05$

Metric	Estimated percent change	95% CI	*p*-value (adj)
Testing	23.31	(− 1.14, 53.46)	0.085
Counseling	24.45	(− 11.08, 75.79)	0.211
Number of new participants registered	29.42	(− 7.10, 84.08)	0.155
Reproductive	56.68	(− 7.10, 160.62)	0.126
Doses of naloxone distributed	57.04*	(23.03, 101.37)	0.009
Total number of unique participants	66.72*	(12.72, 152.93)	0.041
Number of encounters	74.18*	(22.46, 152.93)	0.022
OD education	84.50*	(16.14, 195.80)	0.041
Wound care	131.21*	(54.88, 246.74)	0.009

A total of ten metrics were analyzed for potential effects associated with RAD affiliation, results shown in Table 2. In the sections that follow, we take a deeper look at two of the 10 total metrics that are of particular interest: (1) the total number of encounters per quarter, and (2) wound care. The total number of encounters is a direct indicator of site traffic, which is a fundamental metric related to quantifying engagement with SSPs. Additionally, we investigate wound care, which quantifies the amount of healthcare services related to wound burden, either direct or referred, that the SSP sites are providing to participants.

### Number of encounters

**Fig. 4:**
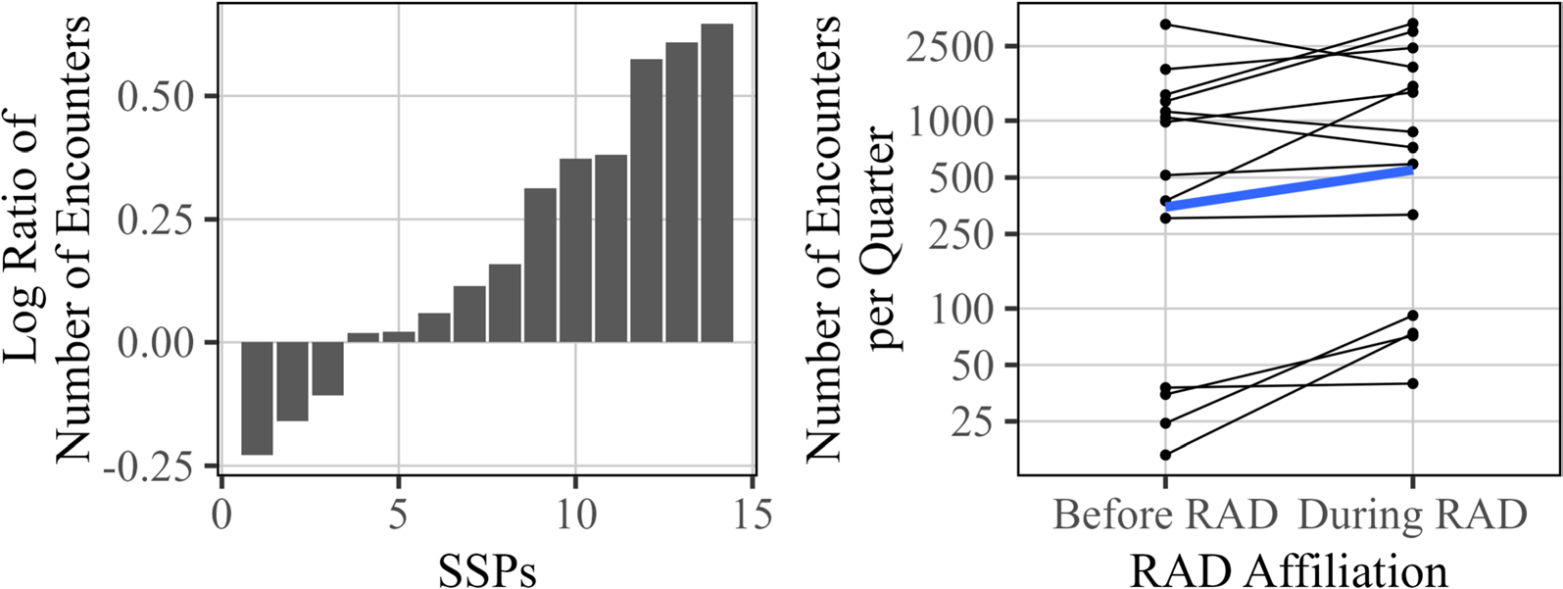
Overall changes in the number of total encounters seen associated with RAD participation. The left plot displays the observed log ratio of the number of encounters during RAD affiliation to the number of encounters prior to RAD affiliation, for each site. The right figure displays the same changes along with the average values before and after RAD affiliation (blue)

For sites that became RAD affiliated, changes in the number of encounters before and after RAD affiliation is displayed in Fig. 4. The left-hand plot shows the log ratio for each of these sites (a log ratio of zero implies no change), and the right-hand plot shows the individual values before and during RAD affiliation for each site. Of the 14 sites that became RAD affiliated by the end of the study duration and that were included in the analysis of encounters, only 3 recorded a decrease in the number of encounters. As shown in Table 2, the estimated change in the number of encounters due to RAD affiliation is around 74% (95% CI 22.5% to 152.9%).

**Fig. 5:**
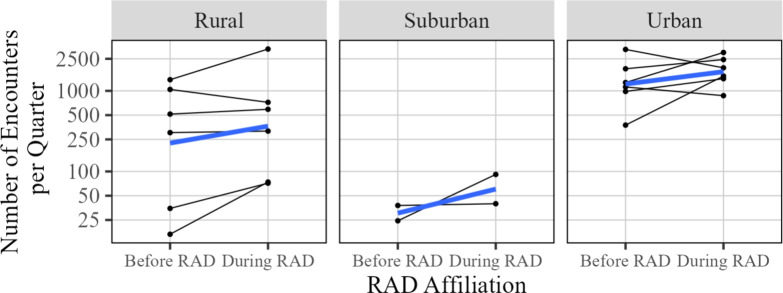
Change in number of encounters, grouped by type of site (rural, suburban, or urban). The average change is shown in blue

To investigate any differences based on location type (rural, suburban, or urban) we display the same changes, but group the results by location type. The designation of rural, suburban, and urban was made in line with the map provided by the Rural Maryland Council in their Fiscal Year 2023 Annual Report [19]. Visually, the changes appear roughly similar across location types, and statistical models similar to (1) accounting for location type do not detect a meaningful difference. It is worth noting that only two sites that became RAD affiliated were in a location classified as suburban, so conclusions are limited for this location type (Fig. 5).

**Fig. 6:**
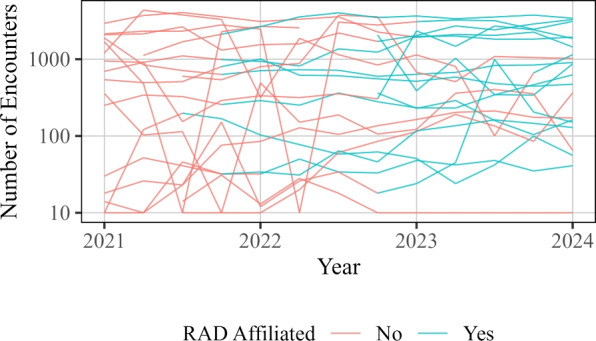
Number of encounters over time. Each thin line represents an individual SSP. Red points and lines indicate data prior to RAD affiliation, and blue points and lines indicate data collected during RAD affiliation

**Fig. 7:**
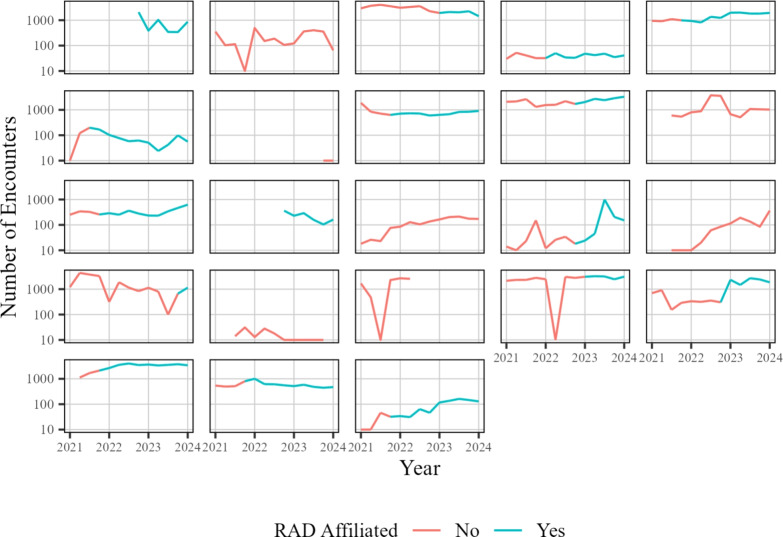
Number of encounters for each quarter across time at each site. The red color indicates a site has yet to become RAD affiliated, whereas blue indicates the data collected is during RAD affiliation

Instead of aggregating the data into pre- and post-RAD affiliation groups, we can also look at the trends over time. In Fig. 6, we display the total number of participant encounters for each quarter across the duration of the collected data. The plot simply mirrors the aggregate analysis: there is variability within and between sites, but overall, there is a moderate but meaningful apparent difference between the data collected during RAD affiliation and data collected prior to RAD affiliation, though this magnitude may vary with time. The individual site data is shown separately for each site in Fig. 7.

### Wound care

**Fig. 8:**
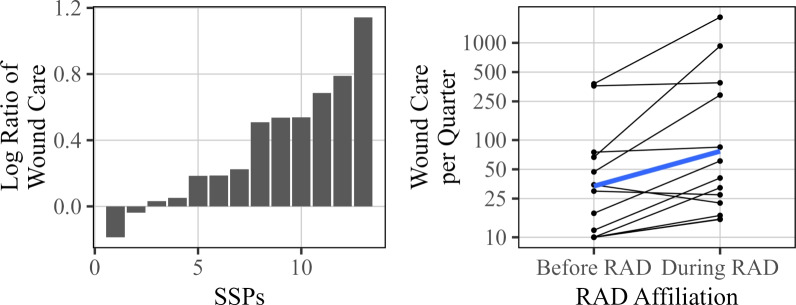
Overall changes in wound care associated with RAD participation. The left plot displays the observed log ratio for each site included for analysis, and the right figure displays the same changes along with the average change (blue) before and after RAD affiliation

**Fig. 9:**
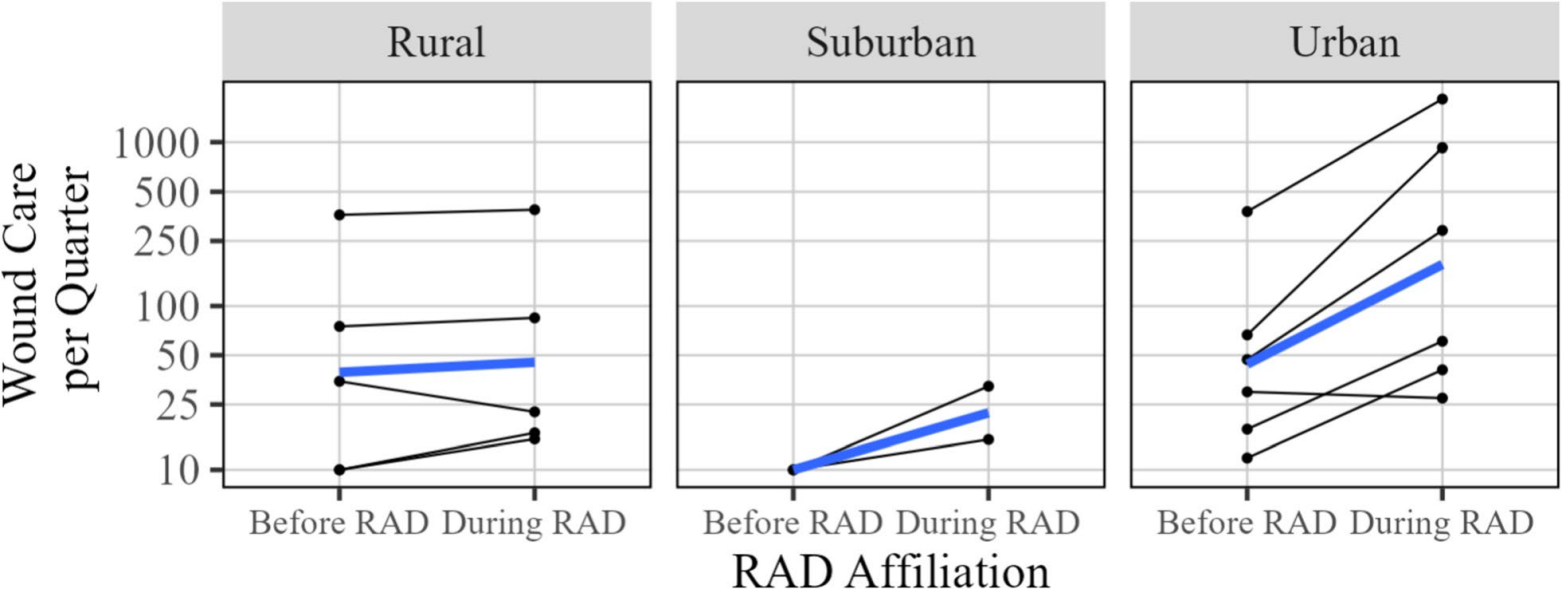
Change in wound care services provided, grouped by type of site (rural, suburban, or urban). The average change for each location type is indicated in blue

The wound care metric represents the number of direct services encounters or referrals to wound care services for visitors to the SSPs. The difference in the amount of wound care services provided before RAD affiliation and after is displayed in Fig. 8. Of the 13 sites that became RAD affiliated by the end of the study duration and were included in analysis of wound care, all but two showed an increase in the number of wound care services provided. These trends are shown across location types (urban, suburban, rural) in Fig. 9, with no apparent substantial differences. As shown in Table 2, the estimated change in wound care due to RAD affiliation is around 131% (95% CI 54.9% to 246.7%). Wound care showed the highest estimated change among all recorded metrics.

**Fig. 10:**
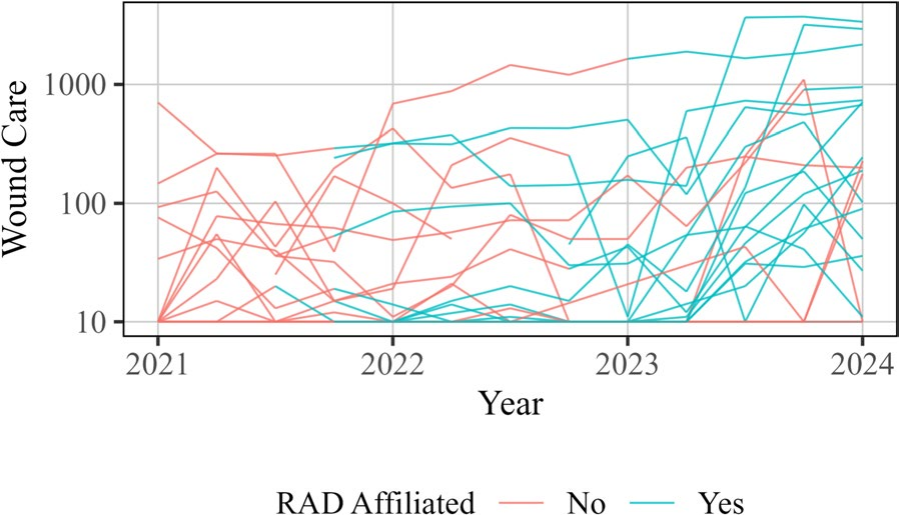
Wound care services provided for each quarter across time at each site. Each thin line represents an individual SSP. Red points and lines indicate data prior to RAD affiliation, and blue points and lines indicate data collected during RAD affiliation

**Fig. 11:**
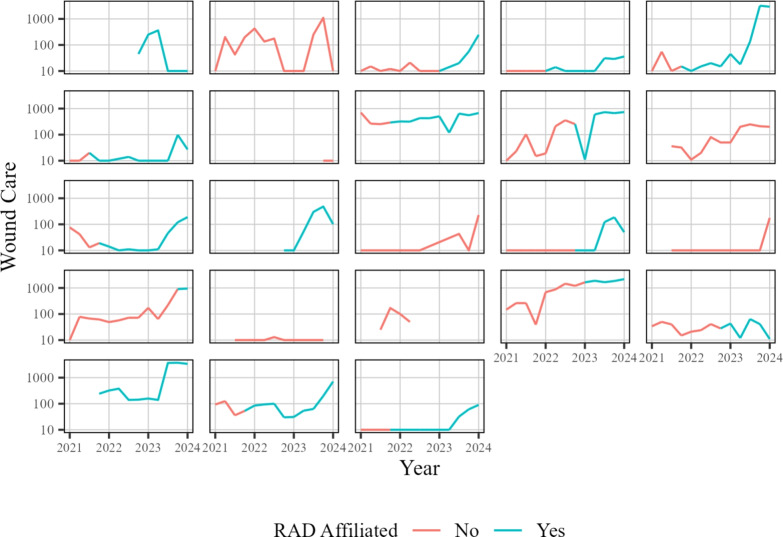
Wound care services provided for each quarter across time at each site. The red color indicates a site has yet to become RAD affiliated, whereas blue indicates the data collected is during RAD affiliation

As was done with the number of encounters, we also look at the trends over time. In Fig. 10, we display the total number of participants seen for each quarter across the duration of the collected data. The individual site data is shown separately for each site in Fig. 11.

### Limitations

**Fig. 12:**
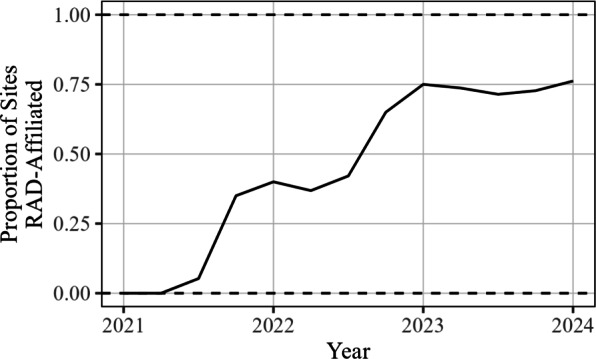
Proportion of sites that are RAD affiliated by quarter for the Wound Care metric

Although the collected data provides an expansive view of the RAD program, there are several aspects of the data that are worth noting. Perhaps most important is that this dataset is observational. Consequently, while RAD affiliation is associated with positive changes, we can’t claim solely from this analysis that RAD is necessarily causing these changes. There may be confounding variables with RAD affiliation, such as SSP maturity (i.e. certain metrics might naturally improve as a site gains more experience), as well as changes in the drug landscape that may be changing over time (e.g. xylazine and medetomidine presence influencing wound care need). RAD affiliation could also be confounded with better site resources such as more staff. Confounding factors that are associated with time are particularly difficult to disentangle using these data, since the vast majority of sites become RAD affiliated at some point during the study, and thus we have relatively few examples of non-RAD site behavior for the later time periods. This limits our ability to attribute observed changes to RAD adoption versus other confounding variables that are correlated with time. This association of time with RAD affiliation can be seen in Fig. 12 below, where the proportion of sites that are RAD affiliated is shown across time (using data from the Wound Care metric). This moderately strong association makes it difficult to isolate the effect of RAD affiliation from time (and thus confounding variables associated with time). Models incorporating both will suffer from the effects of collinearity, making interpretability difficult. We attempt to address some of these challenges in the next section, but we note that claims of causality cannot be made from the data alone, especially given RAD affiliation’s positive association with time.

Despite this limitation, for some metrics, there is reason to suspect that the RAD program is linked with the observed changes, for reasons that exist outside of the data analysis. For example, the information on contaminants and adulterants within provided drug samples may lead participants to be more observant of the development of skin and soft tissue infections and seek out these services in real time given their availability on site. Similarly, a better understanding of the components of sample drugs may make participants more open to receiving naloxone and information on mitigating the risks of overdose.

### Addressing potential confounding variables

#### Adjusting for baseline trends in time

In Sect. 4, we provided analysis quantifying the changes observed across the recorded metrics for the sites that became RAD affiliated. That analysis largely aims at answering the question: “to what extent do the various metrics change when a site becomes RAD affiliated?" While an important question, this does not adjust for any baseline trends seen over time in non-RAD affiliated data, making it potentially difficult to draw conclusions about the effects of RAD on said metrics that could be due to confounding variables such as site maturity. One challenge to investigating this question is the relatively few examples of RAD sites that both (1) did not become RAD affiliated for the duration of the data collection and (2) were not excluded from analysis by the exclusion criteria outlined in Sect. 2. For most metrics, only 6 or 7 sites did not become RAD affiliated by the end of the study duration, so examples of RAD behavior is limited in later time periods. Therefore, it is somewhat difficult to tease out whether trends seen in earlier time periods persist in the same manner into the late time periods. Nevertheless, there are still a handful of sites for each metric available that do not become RAD affiliated. We fit a model that adjusts the effect estimates for time, injecting caution into interpretation. We use the following mixed effects model to estimate the effect of RAD, adjusting for global trends in time, within-site correlation, and the unbalanced nature of the data with respect to RAD affiliation:

2 $$\begin{aligned} \log (Y_{{ij}} ) = & \,\beta _{0} + \beta _{1} t_{{ij}} + \beta _{2} t_{{ij}} I({\mathrm{rad}}_{{ij}} ) + \beta _{3} I({\text{eventual rad}}_{i} ) + b_{{0i}} + \epsilon _{{ij}}\\ & \, b_{{0i}} \sim N(0,\sigma _{b}^{2} );\,\epsilon _{{ij}} \sim N(0,\sigma ^{2} ) \\ \end{aligned} $$

**Fig. 13:**
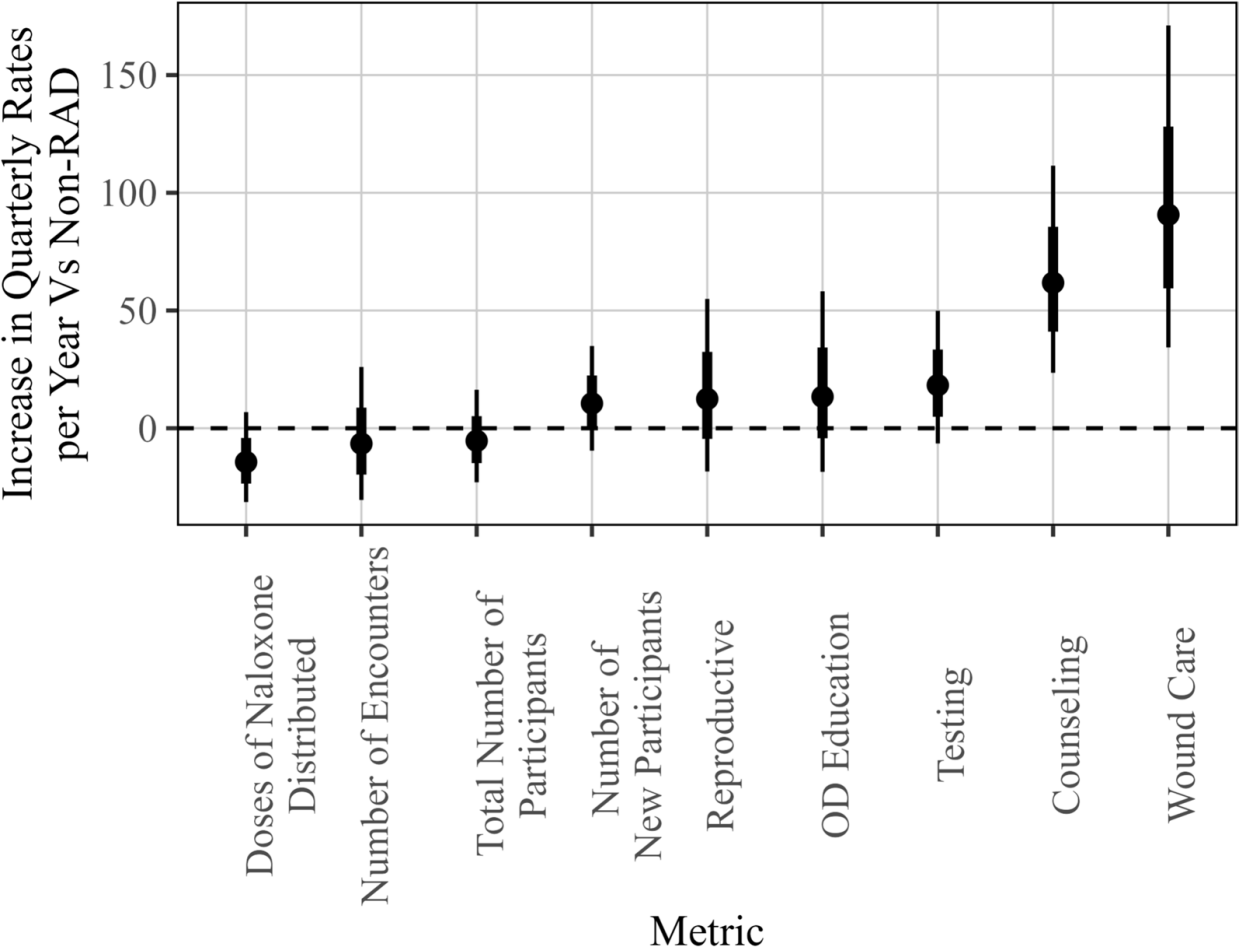
Percentage change in each metric versus non-RAD affiliated sites, as well as standard errors (thick black lines) and 95% confidence intervals (thin black lines)

This model is similar to that of (1), but with a few additional terms. To account for trends over time, the model uses time as a covariate, indicated as *t*, which represents the particular year and quarter from which the observation was made. The indicator variable $I(\text {rad}_{ij})$ represents whether the given measurement is observed during RAD affiliation, and $I(\text {eventual rad}_i)$ represents whether the particular site eventually becomes RAD affiliated. A random effect for intercept, $b_{0i}$, is used to model differences between sites, and to adjust for within-site correlation. (Note: more complex models were considered, e.g., random slopes, but diagnostics suggested these models were over-parameterized with respect to the sample size.) The primary parameter of interest is $\beta _2$, which estimates the additional annual increase (on a log scale) of the given metric associated with being RAD affiliated, beyond the general trend over time for data collected when a site is not RAD affiliated, captured by $\beta _1$. The term $\beta _3$ is included to adjust for any systematic offsets for sites that eventually become RAD affiliated. The estimated results are shown below in Fig. 13.

The results in Fig. 13 show that while many metrics suggest a positive association with RAD affiliation, only Wound Care and Counseling reach statistical significance for this data. Compared to the previous analysis, the effect sizes are generally lower than the estimates given at the beginning of Sect. 4, since here we are adjusting for positive trends seen in data from sites that are not RAD affiliated. The large uncertainties in the estimates are likely due to the amount of variability seen both within and between sites, as well as a relatively small sample size which limits statistical power. Thus, a larger sample size would be needed to distinguish many these effects from zero statistically at a 95% confidence level. Additionally, as mentioned previously, these results should be interpreted with caution, since we have a relatively few number of sites (6 or 7 in most cases) that do not become RAD affiliated, and many sites that become RAD affiliated do so relatively quickly.

#### Changes in policy for purchase of safe smoking supplies

Another potential source of confounding for site traffic is the allowance of Maryland state funds for the purchase of safe smoking supplies. The usage of funds for smoking supplies was prohibited beginning in July of 2021, but then became permitted again in July of 2023. This policy change may have contributed to an increase in site traffic seen in the second half of 2023 and 2024, as the distribution of safe smoking supplies is a popular service provided by SSPs. To investigate whether this data suggests a significant increase in traffic occurring for this time period, we fit the original statistical model (1) with an additional fixed effect term that indicates whether the observed number of encounters occurred after Q2 of 2023. Using the lmerTest R package [20, 21], the estimated change in the total number of encounters that occurred after Q2 of 2023 across all sites is 23%, but this value is not statistically significant. Of course, this time period is somewhat correlated with RAD affiliation, since the change from RAD to non-RAD affiliation tended to occur at later time points, and so these two effects can’t be estimated completely independently. However, the effect for RAD affiliation still persists in this model, and is much larger in magnitude than the increases seen starting in Q3 of 2023 across all sites. This suggests that while the policy change may have led to increases in site traffic, it does not appear to be a significant factor in explaining the magnitude of the increase in traffic seen.

## Discussion

In this paper, we investigate the association of the implementation of the Rapid Analysis of Drugs (RAD) program within Maryland’s Syringe Service Programs (SSPs) on engagement and service delivery, metrics which have been shown to have positive impacts on health outcomes for PWUD [22]. Through an analysis of data from 24 SSP sites across the state, it is evident that RAD affiliation is associated with increases in several key metrics. Notably, in initial analyses comparing pre- and post-RAD metrics, estimates of increases were seen in the number of encounters, which rose by an estimated 74% (95% CI 22.5% to 152.9%) following RAD affiliation, and wound care services, which saw an estimated 131% (95% CI 54.9% to 246.7%) increase. While the confidence intervals on these estimates are broad, they do indicate a statistically significant positive association with these metrics and joining the RAD program. It is worth noting, however, as discussed in Sect. 4.4.1, that these effects may be moderated by the baseline trends seen in non-RAD affiliated data (potentially attributable to factors such as site maturity), though data are somewhat limited for this analysis.

The results discovered related to wound care services in this study are particularly notable. For background, xylazine became a prevalent adulterant in the Maryland drug supply during the study period and the substance has been associated with skin and soft tissue infections among PWUD requiring treatment [23–25]. We can say anecdotally that RAD sampling highlighted the penetrance of xylazine into local drug markets for SSP providers and participants; possibly leading to increased SSP participant surveillance for and identification of wounds as well as their demand for wound care services in general. If SSP staff responded by supplying the requested services, this would certainly lead to the increase in service utilization over time. This would be consistent with the results from Sect. 4.2, where we see a large increase in the use of wound care services pre- to post-RAD affiliation and a positive correlation over time, meaning that the increased utilization of wound care services could be directly attributable to xylazine’s increased prevalence in the Maryland drug supply during the study period across all SSPs- not only those associated with the RAD program. If another substance, not associated with wounds, was readily identified in the drug supply then a different outcome may have occurred. Further, it is impossible to say without further study whether the SSPs (RAD or otherwise) would have been as prepared for the wound care increases without the advanced notice that RAD sampling provides.

These findings may suggest that RAD’s role in providing drug composition data helps build trust with PWUD, empowering them to engage more actively with harm reduction services and seek necessary care, however further research is needed to assess the extent to which participation in RAD has causal impact on particular engagement metrics. Similar to the secondary exchange of supplies commonly seen in SSP work, RAD information is often passed along in the community as well. This information sharing could encourage people in the community who do not necessarily provide drug samples through RAD to also engage in harm reduction services and choose informed risk reduction tactics (like carrying naloxone, not using alone, or using test strips). Study on RAD affiliation’s behavioral effects would be an interesting future direction. The positive associations in metrics seen across various sites, especially in health services like overdose prevention education and wound care, suggests that the RAD program is an interesting subject of further study in enhancing service engagement and outcomes. Despite the promising results, several limitations of the study must be acknowledged, particularly the observational nature of the data, which precludes definitive conclusions about causality. Other factors, such as the maturity of SSP sites and broader changes in the drug landscape, may have contributed to observed changes. And it is important to note that SSP sites who choose adoption of RAD may be better staffed in the first place, though in our analysis we attempt to account for this as much as possible by conducting within-site comparisons. Future research could explore these associations further, accounting for additional confounding variables, incorporating drug supply data, using mixed-methods evaluations to more robustly assess the impact of RAD and long-term sustainability of these improvements. Overall, the RAD program represents a crucial step forward in the public health response to the evolving overdose crisis in Maryland, providing a model for other regions grappling with similar challenges in the fentanyl era.

## Data Availability

The datasets generated and/or analysed during the current study are not publicly available due to protecting the privacy and safety or Syringe Services Programs and their participants but are available from the corresponding author on reasonable request.

## Footnotes

1 The RAD project is part of NIST’s Rapid Drug Analysis and Research (RaDAR) Program (https://www.nist.gov/programs-projects/rapid-drug-analysis-and-research-radar-providing-near-real-time-insight-illicit).
